# Effect of VIRP1 Protein on Nuclear Import of Citrus Exocortis Viroid (CEVd)

**DOI:** 10.3390/biom11010095

**Published:** 2021-01-13

**Authors:** Hyesu Seo, Kyunghee Kim, Woong June Park

**Affiliations:** Department of Molecular Biology, Dankook University, Cheonan-si 31116, Korea; hsuseo89@daum.net (H.S.); kkh1701@daum.net (K.K.)

**Keywords:** viroid, nuclear import, VIRP1, citrus exocortis viroid (CEVd)

## Abstract

Before replicating, *Pospiviroidae* viroids must move into the plant nucleus. However, the mechanisms of viroid nuclear import are not entirely understood. To study the nuclear import of viroids, we established a nuclear import assay system using onion cell strips and observed the import of Alexa Fluor-594-labeled citrus exocortis viroid (CEVd). To identify the plant factors involved in the nuclear import of viroids, we cloned the *Viroid RNA-binding Protein 1* (*VIRP1*) gene from a tomato cultivar, Seokwang, and heterologously expressed and purified the VIRP1 protein. The newly prepared VIRP1 protein had alterations of amino acid residues at two points (H52R, A277G) compared with a reference VIRP1 protein (AJ249595). VIRP1 specifically bound to CEVd and promoted its nuclear import. However, it is still uncertain whether VIRP1 is the only factor required for the nuclear import of CEVd because CEVd entered the plant nuclei without VIRP1 in our assay system. The cause of the observed nuclear accumulation of CEVd in the absence of VIRP1 needs to be further clarified.

## 1. Introduction

Viroids are plant pathogens consisting of a single-stranded non-coding circular RNA molecule with 246–401 nucleotides [1,2]. In contrast to viruses, viroids do not have protein capsids. The secondary structures with loops and stems formed by the base pairing among the nucleotides on the same RNA strand may be stable enough to maintain the viroid activity even without any protein capsid. The use of energy-minimizing prediction models has indicated that pospiviroids, replicating in plant nuclei, have a rod-shaped structure [3,4]. The predicted rod-shaped structure of the potato spindle tuber viroid (PSTVd) has been confirmed by analytical ultracentrifugation [5], selective 2′-hydroxyl acylation analyzed by primer extension (SHAPE) [6,7], atomic-force microscopy [8], and other methods [9]. Five domains—terminal left, pathogenic, central conserved, variable, and terminal right-domain [10]—in the rod-shaped pospiviroids can be distinguished through their structural characteristics and differential contribution to the viroid infection cycle, although details of the pathogenic mechanisms of viroids largely remain undiscovered. It is likely that a certain spatial conformation [9,11,12] in each domain is crucial for viroid activities. Viroids are generally classified into two families: *Pospiviroidae* and *Avsunviroidae*. Avocado sunblotch viroid, an *Avsunviroidae* viroid, has been detected in chloroplasts of infected cells [13], while PSTVd [14] and citrus exocortis viroid (CEVd) [15], members of *Pospiviroidae,* are found in the nuclei.

When *Pospiviroidae* viroids enter plant cells, they move into nuclei, replicate there, exit the nuclei, and move to other parts of the host plants [16]. In studies to find the site of replication, PSTVd [14,17] and CEVd [15,18] were recovered from the nuclei of infected leaves. The nuclear localization of PSTVd [19], coconut cadang-cadang viroid (CCCVd), and CEVd [20] was imaged with high-resolution-microscopy following in situ hybridization in tomato plants. Because *Pospiviroidae* viroids are associated with plant RNA polymerase II (RNA Pol II) [21], which generally transcribes protein-coding genes, and the DNA-dependent RNA Pol II was shown to produce viroids in vitro [22], viroid replication is thought to occur in plant nuclei via RNA Pol II. The transcription factor IIIA (TFIIIA), which specifically binds to PSTVd [23], is an axillary factor for the RNA Pol II-dependent PSTVd replication [24]. There are two forms of TFIIIA that have either seven (TFIIIA-7ZF) or nine (TFIIIA-9ZF) zinc finger domains [24]. Although both TFIIIA-7ZF and TFIIIA-9ZF bind to PSTVd, TFIIIA-7ZF is the main contributor to PSTVd replication [24]. Viroids need to hijack host proteins for replication because their RNA does not encode proteins. Furthermore, such use of host proteins is common in viral infection cycles [25].

The nuclear import of PSTVd was found to be specifically competitive, and insensitive to GTP-γ-S and cytoskeleton-disturbing chemicals [26]. Studies showed that the (+)-strand of the viroid accumulates in the nucleolus, as well as in the nucleoplasm, whereas the (-) strand remains in the nucleoplasm [27]. However, details of the viroid nuclear import mechanism remain largely uninvestigated. As viroids replicate using host proteins, they may move into plant nuclei also utilizing host protein factors. Any host protein with viroid-binding activity is a reasonable candidate for components of the cellular machinery that is hijacked by viroids for their nuclear import.

Silencing of the gene coding the plant-specific 4/1 protein changed the accumulation and transport of PSTVd [28]. Plant-specific 4/1 protein has been known to interact with viral movement proteins [29] and contribute to the transport of macromolecules through plasmodesmata [30]. Despite the existence of both a nuclear export signal [31] and nuclear localization signal (NLS) [32] in plant-specific 4/1 protein, the role of this protein in viroid nuclear import is unclear.

Viroid RNA-binding Protein 1 (VIRP1) from tomato plants has a bromodomain [33] and is located in the nucleus [34]. VIRP1-silenced plants are tolerant to PSTVd and CEVd, indicating the important role of VIRP1 in viroid infection. A structural motif, the RY motif, at the terminal right loop is critical for the binding of viroid to VIRP1 [35]. Interestingly, VIRP1 (also called as bromodomain-containing protein 1) mediates the nuclear import of the satellite RNA from cucumber mosaic virus [36]. We established a nuclear import assay system using onion cell strips and confirmed that the system functioned well. Utilizing the assay system, we investigated the nuclear import of CEVd and evaluated the effect of VIRP1.

## 2. Materials and Methods

### 2.1. Labeling of CEVd with Alexa-Fluor-594 by In Vitro Transcription

The DNA fragment corresponding to the linear monomeric CEVd (Access No. M34917) [37] was constructed by applying a PCR-based method [38], cloned in pGEM-T Easy Vector (Promega, Madison, USA) and confirmed by sequencing (Kim et al., in preparation). CEVd was prepared by in vitro transcription after the plasmid vector was cut with *Eco*RI (Takara, Kusatsu, Japan) to provide a proper termination point. To prepare the Alexa-Fluor-594-labeled CEVd (red fluorescence), the CEVd-containing plasmid vector (1 μg) was transcribed in vitro with 100 units of T7 polymerase (New England Biolabs, Ipswich, USA) in the presence of 200 μM of ChromaTide^TM^ Alexa-Fluor-594-5-dUTP (Invitrogen, Waltham, USA) in a 30-µL reaction mixture at 37 °C for 3 h, following the manufacturer’s instructions. The reaction was stopped by treating the mixture with 2.5 units of RNase-free recombinant DNase I (Takara, Kusatsu, Japan) at 37 °C for 20 min. Finally, the Alexa-Fluor-594-labeled CEVd was purified with NucAway Spin Columns (Invitrogen, Waltham, USA).

### 2.2. Preparation of VIRP1 Protein

Total RNA was isolated from 20-d-old tomato (*Solanum lycopersicum* cv. Seokwang) leaves and reverse transcribed with M-MLV (Promega, Madison, USA). The cDNA was used to amplify the full-length coding sequence of the *VIRP1* gene (AJ249595) [33] by PCR, using a VIRP1-specific forward primer (AAA GAA TTC ATG GCA TCC GCC GTC TTA GC) with an *Eco*RI site and a reverse primer (AAA CTC GAG AGA GTG TGC ATC ATC AGC ATC AG) containing a *Xho*I site. The DNA fragment amplified by PCR using a high fidelity Taq polymerase (TaKaRa Ex Taq, Takara, Tokyo, Japan) was cloned into a pET28a expression vector containing a C-terminal 6xHis-tag (Invitrogen, Waltham, USA), and the cloned gene was confirmed by sequencing. *Escherichia coli* strain BL21 was transformed with the completed expression vector, and the expression of VIRP1 was induced with 0.1 mM (isopropyl β-D-1-thiogalactopyranoside (IPTG) at 30 °C for 3 h. The induced cells in 1 L of Luria-Bertani (LB) medium were harvested by centrifugation at 5400× *g* for 5 min at 4 °C (LABOGENE, Seoul, Korea). The pellet was resuspended in 9 mL of ice-cold 1 × STE buffer and treated with 10 mg/mL of lysozyme (USB Biochemicals, Cleveland, USA) for 30 min in the presence of 3% phenylmethylsulfonyl fluoride (PMSF) (Sigma-Aldrich, St. Louis, USA) in 10 mL of the final mixture. The sample volume was increased with 10 mL of 1 × STE buffer, and the sample was then sonicated (Vibra-Cell Ultrasonic Liquid Processors, Sonics, Newtown, USA) for 10 s on ice, and this was repeated 60 times with cooling intervals. The sonicated preparation was then treated with 1% Triton X-100 (bioWORLD, Dublin, USA) for 20 min on ice and cleared by centrifugation (19,500× *g* 20 min, 4 °C). The supernatant was collected and subjected to the following purification steps. VIRP1 protein was purified from the crude extract with a Ni-NTA column (Clontech, Tokyo, Japan) following the manufacturer’s instructions. The final fraction containing 65.6 kDa protein was obtained by a native electrophoretic separation method (Model 491 Prep Cell, Bio-Rad, Hercules, USA). The Ni-NTA-purified and size-fractionated VIRP1 protein was concentrated with Amicon Ultra-15 Centrifugal Filter Units (Merck, Darmstadt, Germany). The final sample was resolved by SDS-PAGE to confirm the purity and the size of the protein.

### 2.3. Nuclear Import Assay Using Onion Cell Strips

A fresh bulb of onion (*Allium cepa*) was cut with a razor blade and thin cell strips were isolated. Pieces of onion cell strips (3 × 4 mm) were floated on and permeabilized with 0.2% Triton X-100 in import buffer (20 mM HEPES, pH 7.4, 100 mM potassium acetate, 2 mM magnesium acetate, 1 mM EGTA) following Merkle et al. [39] for 55 min on ice. The permeabilized cell strips were washed with import buffer without Triton X-100 and used for the nuclear import assay. The permeabilized pieces of onion cell strips were incubated with test materials. To check whether the cells were permeabilized, the cell strips were incubated with 0.3% rhodamine B isothiocyanate-dextran (70 kDa) (Sigma-Aldrich, USA) in import buffer for 150 min at room temperature. To test the integrity of the nuclear membrane after the permeabilization treatment and to determine whether the nuclear import system worked properly, 0.4 μg/μL of mCherry::mCherry protein (53.4 kDa) or NLS::mCherry::mCherry (NLS from SV40) proteins were added to the permeabilized onion cell strips for 140 min at RT. To observe the nuclear accumulation of CEVd, Alexa Fluor 594-labeled CEVd was supplied to onion cell strips (0.02 μg/μL Alexa Fluor 594-labeled-CEVd RNA, 20 mM GTP, 1 mM ATP, 10 mM creatine kinase, 50 μg/mL creatine phosphate, 0.3 μg/μL wheat germ extract in 30 μL 1x import buffer). Depending on the purpose of the test, 0.4 μg/μL of VIRP1 was also added to the import buffer just before the start of the assay. When the import process was stopped, the cell strips were fixed with 0.36% formaldehyde for 20 min and washed twice with import buffer. The fluorescent images were observed under a confocal microscope (LSM 700, Zeiss, Oberkochen, Germany) with a transmitted light detector. The nuclei in the cells were stained with 4′,6-diamidino-2-phenylindole (DAPI) at a concentration of 10 μg/mL. The fluorescence signal in the nuclei was quantified with ImageJ (https://imagej.nih.gov/ij/).

### 2.4. Electrophoretic Mobility-Shift Assay (EMSA)

CEVd was labeled with digoxigenin (Dig) by in vitro transcription in the presence of Digoxigenin-11-UTP (Roche, Basel, Swiss) following the protocols supplied by the manufacturer. The Dig-labeled CEVd and purified VIRP1 protein were mixed and incubated in the binding buffer (20 mM HEPES-NaOH, pH 8.0, 50 mM KCl, 100 μM EDTA) in the presence of 1 μg/μL of yeast tRNA (Invitrogen, Waltham, USA) for 1 h at 22 °C. The mixed sample was resolved on a non-denaturing 6% polyacrylamide gel (29:1) in 0.5 × TBE at 70 V. The resolved materials were transferred to a sheet of nylon membrane (Nytran SuPerCharge, Schleicher & Schuell Biosciences, Dassel, Germany) in the flow of 10 × SSC buffer. The transferred materials were cross-linked to the nylon membrane by UV illumination (30,000 J/cm^2^) in an ultraviolet cross-linker (Hoefer-UVC 500, Amersham Biosciences, Little Chalfont, UK). The Dig signal on the membrane was detected by following the manufacturer’s instructions (Roche, Basel, Switzerland). Briefly, the fixed membrane was washed with 1 × maleic acid buffer (100 mM, pH 7.5) and blocked with 1 × blocking buffer (Roche, Swiss) containing 0.5% TWEEN 20 (Sigma, USA) for 30 min at 27 °C in a hybridization oven with a rotating wheel (Combi-12, FINEPCR, Seoul, Korea). Then, the membrane was incubated with anti-Dig polyclonal antibodies (0.1 μg/μL) conjugated with horseradish peroxidase (HRP) (Abnova, Taipei, Taiwan) for another 30 min at 27 °C. The membrane was washed twice with maleic acid buffer (pH 7.5) containing 0.5% TWEEN 20. It was then equilibrated with the alkaline (pH 9.5) detection buffer (Roche, Basel, Swiss) containing 0.5% TWEEN 20. HRP activity was detected with West-Zol plus (iNtRON Biotechnology, Seoul, Korea) and recorded with an image analyzing system (ChemiDoc XRS+, Bio-Rad, Hercules, USA).

## 3. Results and Discussion

### 3.1. Establishment of Nuclear Import Assay System Using Onion Epidermal Cell Strips

**Figure 1:**
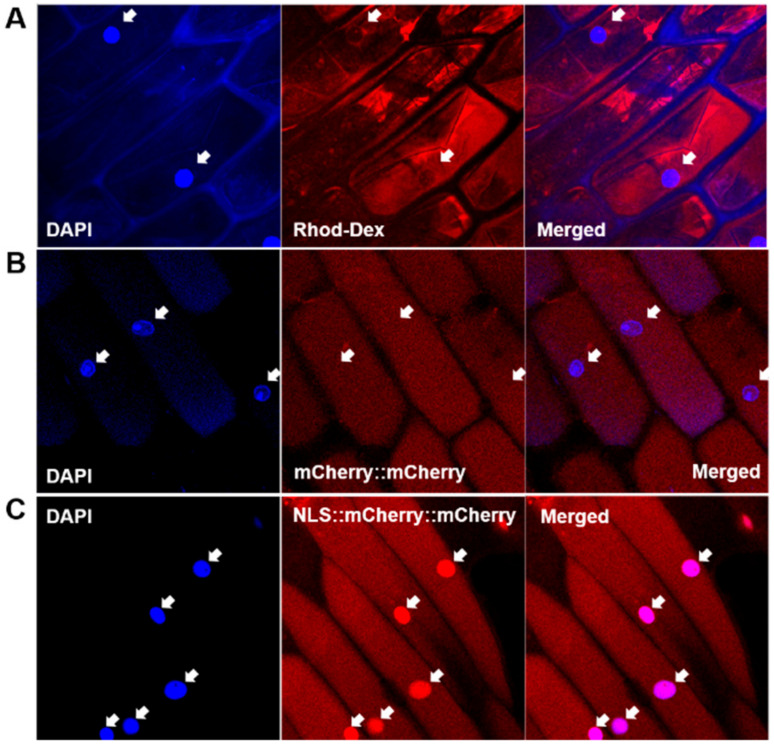
Establishment of nuclear import assay system using onion cell strips. (**A**) Cytoplasmic accumulation of rhodamine-dextran (70 kDa) after permeabilization of plasma membrane with 0.2% Triton X-100. (**B**) Cytoplasmic accumulation of mCherry::mCherry protein (53.4 kDa). (**C**) Cytoplasmic and nuclear accumulation of nuclear localization signal (NLS)::mCherry::mCherry protein. Arrows indicate the location of nuclei.

We established a nuclear import assay system based on onion cell strips permeabilized with Triton X-100. Exogenously applied rhodamine-labeled dextran passed through the plasma membrane and accumulated in the cytoplasm (Figure 1A). The rhodamine signal was clearly observed in the cytoplasm but not in the nuclei of the permeabilized cells (Figure 1, arrows). This indicated that the plasma membrane was properly permeabilized to allow the macromolecule access and that the nuclear membrane was not affected by Triton X-100.

When tandemly repeated mCherry protein [40] (mCherry::mCherry; MW 53.4 kDa) was supplied to permeabilized onion cells, red fluorescence was observed in the cytoplasm (Figure 1B), again indicating the permeabilization of the plasma membrane and the integrity of the nuclear membrane. When NLS originating from SV40 [41], PKKKRKV, was introduced to the mCherry protein construct, the recombinant mCherry protein (NLS::mCherry::mCherry) was concentrated in the nuclei (Figure 1C). This result shows that the protein nuclear import machinery utilizing the NLS worked properly in our assay system.

### 3.2. Preparation of VIRP1 and Confirmation of VIRP1–CEVd Binding

**Figure 2:**
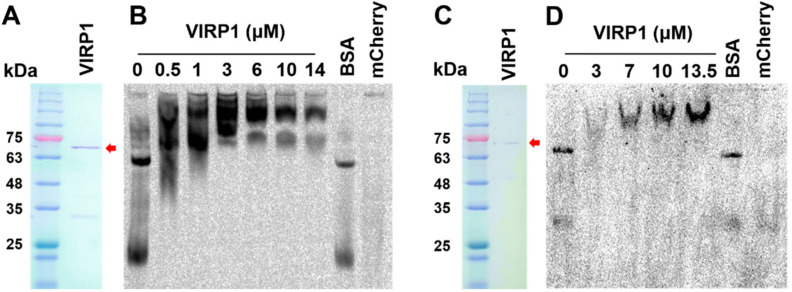
Purification of viroid RNA-binding protein 1 (VIRP1) and its binding to citrus exocortis viroid (CEVd). (**A**) Ni-NTA-purified VIRP1. (**B**) Electrophoretic Mobility-Shift Assay (EMSA) of CEVd with Ni-NTA-purified VIRP1. The CEVd concentration was 50 nM, and the concentrations of bovine serum albumin (BSA) and mCherry were 10.5 µM and 14 µM, respectively. (**C**) Size-fractionated VIRP1 after Ni-NTA purification. (**D**) EMSA of CEVd with size-fractionated VIRP1. The CEVd concentration was 40 nM, and the concentrations of BSA and mCherry were 12 µM and 13.5 µM, respectively. CEVd was labeled with digoxigenin (Dig). mCherry indicates mCherry::mCherry protein with 6xHis tag. Arrows indicate VIRP1. (**A**,**C**): Coomassie-stained SDS-PAGE gels. (**B**,**D**): The Dig signal from the labeled CEVd was developed after transfer to Nylon membrane from a 6% non-denaturing polyacrylamide gel, as described in the Materials and Methods. Bands of CEVd in the absence of VIRP1 may represent different thermodynamically metastable forms [42] as also observed during EMSA in previous reports [33,43].

Using RT-PCR, we amplified the full-length coding sequence of VIRP1 (AJ249595) from the total RNA obtained from tomato leaves and cloned the cDNA fragment in a modified pET28a vector with C-terminal 6xHis-Tag. Although the PCR primers were designed based on the nucleotide sequences of *VIRP1* gene (AJ249595), the cloned sequences revealed changes in two amino acid residues (H52R, A277G). However, the characteristics of the amino acid residues were the same as those of the amino acid residues of AJ249595. Therefore, it was vital to examine the binding activity of the newly prepared VIRP1 before investigating its effect on the CEVd nuclear import. We purified the recombinant VIRP1 protein using a Ni-NTA column (Figure 2A) and further resolved the protein on preparative native PAGE (491 Prep Cell, BioRad, Hercules, USA) to remove small co-purified proteins (Figure 2C). The expected size of VIRP1 was 66.7 kDa. However, proteins with smaller molecular weights were observed after the purification with Ni-NTA. The smaller proteins appeared later, even in the further fractionated samples obtained from 491 PrepCell, when the concentration steps using Amicon Ultra-15 Filter Units (Merck, Darmstadt, Germany) were delayed, suggesting that the small bands observed after the Ni-NTA purification were fragmentation products from VIRP1.

To evaluate the ability of VIRP1 to bind CEVd, CEVd–VIRP1 interactions were analyzed by EMSA with the purified VIRP1 and Dig-labeled CEVd. A band shift, indicating the binding of VIRP1 to CEVd, was observed following the reactions with Ni-NTA-purified VIRP1 (Figure 2B) and the further fractionated VIRP1 (Figure 2D). By adding VIRP1, the free CEVd signal observed in the control gradually disappeared (Figure 2B,D). Because VIRP1 was purified using the C-terminal 6xHis tag, we also tested the influence of the mCherry::mCherry protein, which also has 6xHis tag. The mCherry construct smeared but did not shift the CEVd bands.

We used competition experiments to confirm the specificity of the CEVd–VIRP1 interaction. The addition of a 30 to 200 molar excess of unlabeled CEVd as a competitor to the binding mixture reduced the signals corresponding to the labeled CEVd–VIRP1 complex (data not shown). This showed that the newly prepared VIRP1 specifically bound to CEVd, even with the amino acid residue changes at two points. The EMSA patterns observed with Ni-NTA-purified VIRP1 (Figure 2B) and the further fractionated VIRP1 (Figure 2D) looked slightly different in terms of complexity. The complex pattern shifts with Ni-NTA-purified proteins (Figure 2B) were likely to be caused by the small proteins generated by the fragmentation of VIRP1. The fragmented VIRP1 appeared to retain CEVd-binding activity. The C-terminal part of VIRP1 retains its binding activity to PSTVd [33].

### 3.3. Nuclear Accumulation of CEVd and Influence of VIRP1 Protein

**Figure 3:**
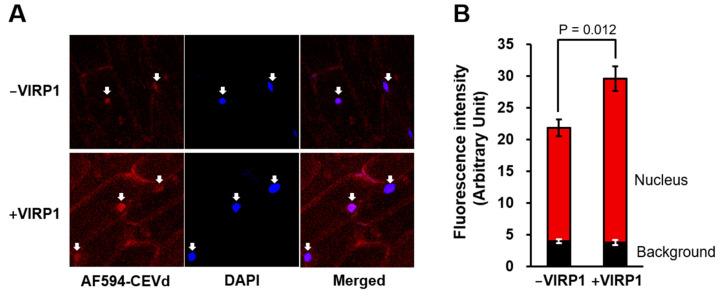
Nuclear import of Alexa Fluor-594-labeld CEVd (AF594-CEVd) and influence of VIRP1 on nuclear import of CEVd. (**A**) Fluorescence images of AF594-CEVd in absence or presence of VIRP1. Arrows indicate the location of nuclei. (**B**) Quantitatively measured nuclear accumulation of AF594-CEVd. Vertical bars indicate the standard errors of four independent experiments. Statistical significance was examined by the Student’s T-test.

The red fluorescent Alexa Fluor 594-labeled CEVd (AF594-CEVd) passed through the plasma membrane and concentrated in the nucleus (Figure 3) during the nuclear import assay with cells from a non-host plant, the onion. Because the integrity of the nuclear membrane was maintained in the permeabilized cells (Figure 1A), the labeled CEVd must have passed through the nuclear membrane via nuclear pores. However, it is unlikely that CEVd passed through nuclear pores by free diffusion. The nuclear import of viroids is essentially specific and competitive [26,44]; therefore, we rejected the possibility of free diffusion and trapping of viroids in the nucleoplasm. The molecular weight of CEVd is more than 110 kDa; thus, it is difficult to imagine that the viroid could freely pass through the nuclear pores, which generally permit the passage of particles less than 40 kDa. Unincorporated Alexa Fluor-conjugated 5-dUTP did not accumulate in the plant nucleus (data not shown).

It has been assumed that viroids use or are helped by host proteins to enter the plant nucleus [44]. We postulated two requirements for the hypothesized host factors involved in viroid nuclear import: viroid-binding activity and nuclear localization. If any plant protein was known to possess both features, it would be a good candidate for a participant in the nuclear import of viroid-like RNA. Some plant proteins with viroid binding-activity have been reported, e.g., plant specific-4/1 protein [28], L5 and TFIIIA [23], and VIRP1 [33], which are all nuclear proteins or proteins with NLS. VIRP1 facilitates the nuclear import of satellite RNA from cucumber mosaic virus [36] and plays important roles in infection by PSTVd or CEVd [44]. Therefore, we tested the effect of VIRP1 on the nuclear import of CEVd.

When the purified VIRP1 protein was mixed with the import buffer, the nuclear import of AF594-CEVd was significantly increased by 44.8% (*P* < 0.05) (Figure 3B). Because most previous studies involved the detection of viroids in plant nuclei by in situ hybridization, the signals from the imported and the replicated viroids were detected together, making it difficult to selectively show the nuclear import. In this study, we showed the promotion effect of VIRP1 on the nuclear import of CEVd. However, it should be noted that AF594-CEVd was transported into the nucleus in the absence of VIRP1 (Figure 3). Furthermore, the nuclear import of CEVd was slightly promoted by the supplementation of VIRP1; in contrast, the nuclear import of the satellite RNA of cucumber mosaic virus was completely dependent on VIRP1 [36]. Thus, VIRP1 may not be the only factor used by viroids to enter the nuclei, and other proteins not investigated by this study may be involved in the nuclear import of CEVd. Therefore, the search for other factors is an interesting focus for further study. Moreover, the endogenous role of VIRP1, in addition to the binding of external RNA molecules, like viroids, is also unclear. The question of whether plant RNAs are transported into the nucleus with the help of VIRP1 remains unanswered.

## 4. Conclusions

VIRP1 protein originating from a tomato cultivar Seokwang was found to have amino acid residue changes at two sites (H52R, A277G) compared with the published one (AJ249595). However, the properties of the two amino acid residues were unchanged, and VIRP1 specifically bound to CEVd as expected. The nuclear import of fluorescently labeled CEVd was observed in an assay system based on onion cell strips. When the purified VIRP1 was added to the nuclear import assay system, the nuclear import of CEVd was significantly increased. However, it is unclear whether VIRP1 is a unique factor involved in the nuclear import of CEVd, as the nuclear accumulation of CEVd was observed in the absence of VIRP1.

## Data Availability

Data are essentially contained within this article.
